# A Survey on Personal Data Cloud

**DOI:** 10.1155/2014/969150

**Published:** 2014-08-05

**Authors:** Jiaqiu Wang, Zhongjie Wang

**Affiliations:** School of Computer Science and Technology, Harbin Institute of Technology, Harbin 150001, China

## Abstract

Personal data represent the e-history of a person and are of great significance to the person, but they are essentially produced and governed by various distributed services and there lacks a global and centralized view. In recent years, researchers pay attention to Personal Data Cloud (PDC) which aggregates the heterogeneous personal data scattered in different clouds into one cloud, so that a person could effectively store, acquire, and share their data. This paper makes a short survey on PDC research by summarizing related papers published in recent years. The concept, classification, and significance of personal data are elaborately introduced and then the semantics correlation and semantics representation of personal data are discussed. A multilayer reference architecture of PDC, including its core components and a real-world operational scenario showing how the reference architecture works, is introduced in detail. Existing commercial PDC products/prototypes are listed and compared from several perspectives. Five open issues to improve the shortcomings of current PDC research are put forward.

## 1. Introduction

With the flourish of cloud computing, especially of the mobile computing technologies, available services on the Internet are drastically increasing and promote people's daily life into a “*service-centric*” style. In the process of service delivery, a great variety of heterogeneous personal data are produced continuously. This phenomenon is in accordance with the growing trend of “big data” in recent years. Initially, data is generated mostly by business information systems in massive organizations and enterprises; along with the flourish of web 2.0, more and more “User Generated Content (UGC)” emerges, and today, a good deal of sensor-based data are automatically collected and aggregated with the help of the Internet of Things (IoT). To sum up, the data generation styles have gone through three phases, that is, passive, voluntary, and automatic [1]. Especially for the last two phases, personal-centric data has become the principal part of “big data”: massive users produce personal data by various Social Network Services (SNS), mobile terminals, and sensors [2].

In recent years, researchers pay attention to the issue of personal data management, in which the effective personal data management across services is a top priority. Efraimidis et al. [3] defined personal data as “the data created by the user or any data about individual,” including (1) user own data created by himself, such as social networking profile; (2) monitoring data, such as location data collected by GPS sensors in his mobile phone; (3) inferred data deduced from the own data and monitoring data, for example, a person's credit score from his transaction records. Kolter et al. [4] listed various types of personal data scattered in a variety of distributed clouds, for example, e-mail and photos stored in a web server and SMS service data stored in the mobile phone. Many other literatures have defined the personal data; however, existing works gave the definition usually by exhaustively listing all types of personal data but there lacks a conceptual one, thereby not being able to cover all types of personal data, especially in today's situation that new services emerge faster and the personal data produced by the new services is more diversified correspondingly.

We consider personal data as any data that is related to a person, not only the data produced by the person himself, but also a (software) service or a device produced data in which the identification of a person is contained. Examples of the former are documents a person drafts, videos and pictures he took, and so forth. The latter covers more broad scope, for example, a professor's salary information produced by his university's human resource service, a patient's health record produced by his wearable devices and medical devices of a hospital, and a traveler's air travel records produced by the online booking services of different airlines. If we consider the person himself as a “*human service*,” the former type of personal data may be regarded as a special one of the latter. Here we give a uniform definition:

**Definition 1 .**

Personal Data is the data produced by any services (not only software and web-based services, but also human and device-based services) around a person during the interactions between the person and the services, on condition that if the person's identification is removed from the data, the data will become meaningless.

**Figure 1:**
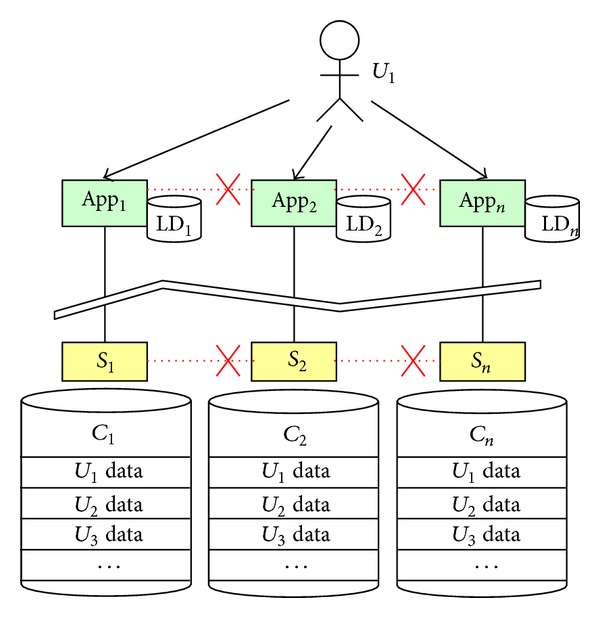
Service-centric personal data management.

Due to the heterogeneity and distributedness of personal data, personal data management exhibits very different characteristics compared with enterprise data or service data management. For the moment, most of the personal data is governed by the provider of the service that produces the data and the data is stored in the cloud of the provider. If a person uses twenty services in his daily life, his personal data is consequentially distributed among twenty logically independent clouds. The person who essentially owns the data has limited privileges only in each service domain but no full authorities to share his personal data across the boundaries between different services. In this case, the person could never get a unified global view on his own personal data. This deprives the right of users as the owner of their data. Especially, in most cases, a person's daily requirements will span across multiple services, and the isolation of his person data hinders the possibilities of autonomic collaborations among services around him.  Figure 1 shows such service-centric personal data scenario.

**Figure 2:**
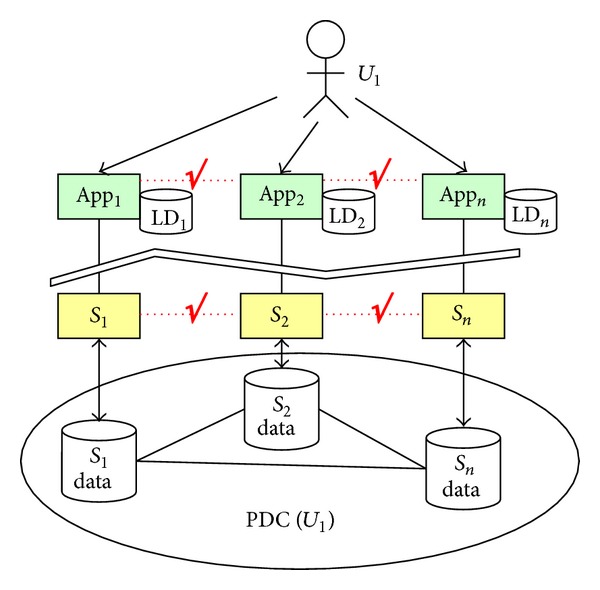
PDC: User-centric personal data management.

To address this challenge, some researchers proposed an idea called “*Personal Data Cloud (PDC)*” to collect and store the personal data of a user in a centralized cloud. PDC, a SaaS application deployed on a specific PaaS platform, plays the role of personal data management in a holistic way. Ideally, services around a person will send the generated personal data directly to his PDC; if not, a dynamic personal data collection component is required to facilitate the synchronization between the cloud of services and PDC of the user. We call it a “*user-centric*” model shown in Figure 2.

In Figures 1 and 2, App_*i*_ refers to an application deployed on the mobile terminals or accessed via web browsers, *S*_*i*_ is a software service deployed on a cloud or a physical service delivered by a device, and the cylinders are the data storages in the clouds. In the service-centric scenario, each service is connected to its own cloud and the personal data of multiple users of this service is stored in the same cloud. Comparatively, in the user-centric scenario, the cloud belongs to the user himself and all his personal data (no matter which service produces the data) are stored in his PDC. The advantage is evident: the isolations among different services are broken and the inherent relations between the user's personal data are recovered and governed in the PDC. Under the support of PDC, the collaborations between services around a person become possible.

This viewpoint is widely endorsed by literatures. Mun et al. [5] thought that “*user-centric*” is the* gravity* shift of information management from organizations to individuals. Technically, this scenario stores personal data in one central core, with domain-specific services plugged into the core, and it is the user that owns the full authority of controlling his data. Ardissono et al. [6] put forward the concept of* personal cloud*, an infrastructure providing an abstraction level over various individual applications and services. Being a unified data management environment, the personal cloud offers complementary functions instead of just linking separate applications and workspaces. Kirkham et al. [7] also proposed a similar idea and believed that such centralized personal data cloud would effectively enable service collaboration around users.

As this is an emerging issue in both research and practice, this paper makes a brief survey on PDC by making an elaborate analysis and summary of related literatures. The objective is to give service and cloud computing researchers/practitioners a global view about the latest research and development on PDC. Remainder of this paper is organized as follows.  Section 2 gives the classification and representation of personal data. A reference architecture of PDC (including its primary components) is introduced in Section 3.  Section 4 lists some existing commercial PDC products/prototypes and compares them from seven perspectives. In Section 5, some open issues about PDC are discussed.  Section 6 concludes the paper.

## 2. Background of Personal Data

### 2.1. Value and Significance of Personal Data

The value and significance of personal data have been fully recognized.

1. Personal data is a partial representation of personal Internet footprint which gradually grows along with a person's daily usage of various services and mobile devices over a period of time. Website accessing records, keywords entered in a search engine, browsing history in an e-Business website, and so on all belong to the personal footprints [8]. These types of data are unintentionally generated by users but are carefully tracked and recorded by search engines and service providers. Analytical tools for recording, aggregating and analyzing the footprints for deep understanding on user behaviors, for example, NM Incite, Social Mention, SocMetrics, Traackr, and Tweepi [9], have been widely adopted.
2. Personal data is a partial representation of personal e-history. In the electronic age, people's daily life is full of intensive interactions with various services, and the generated personal data constitutes his e-history which is always growing. How many cities have I visited in my past life? What kinds of books have I bought from multiple online bookstores? How much investment income have I attained from four banks in the last five years? There are many such questions, but they are all difficult to be answered quickly and accurately. Having effective personal data management, people possess the ability of reviewing and summarizing their own history from various perspectives [10].
3. Personal data is a partial representation of personal habits and preferences. Similar to the footprint, personal habits and preferences are prolifically embedded in personal data, too [11], for example, my favorite books/music, my wish list, my comments on some topics, my blogs, and so forth [12]. Without PDC, each service provider is responsible for collecting such data and analyzing user habits and preferences to make accurate service recommendations but is limited to only one service domain. If PDC was used, such analysis could cover the full-scale personal data from various services and will be more effective and precise.
4. Personal data enables completely personalized service collaboration. Traditional service collaboration is usually dominated by service brokers in a public service platform (e.g.,* eBay*'s e-commerce platform and* Expedia*'s online travel platform), but this way usually offers standard collaboration patterns with the limited degree of personalization. If PDC exists, the personal data represents a user's history and preferences (namely, his personalized requirements on services); therefore, it is easy to conduct the completely personalized collaborations between these services, and the service brokers are no longer necessary [7, 13]. Some commercialized services such as* ifttt.com* and* Google Now* also support the personalized service collaborations in a* user-centric* way instead of traditional* broker-centric* one.

### 2.2. Features of Personal Data

The following four distinct characteristics jointly differentiate the personal data from other types of data.

1. *High degree of dispersion*: referring to the fact that the personal data is scattered in a wide range of IT environments (clouds, mobile devices, etc.) throughout the hardware and software and a variety of service providers [13, 14], thereby it is difficult for data owners to uniformly manage their personal data.
2. *High degree of heterogeneity*: referring to the fact that personal data is composed of a variety of morphologies, with different data types and granularities, and above all, with different semantics representations. The corresponding challenge is the syntax and semantics unification [15].
3. *High degree of correlation*: referring to the fact that there are close correlations between different parts of personal data which are originally stored independently and isolated with each other. This is because these data describe the person's life from different aspects, and such correlations do not depend on where they are stored and who they are managed. To recover such intrinsic correlations, ontology and Linked Data are frequently adopted to correlate the personal data released on the web by using URI and RDF [16].
4. *High degree of privacy*: referring to the fact that personal data should be shared in a strict and limited scope with other people/services. Personal data is vulnerable to be attacked, and excessive openness will result in a lot of privacy and security problems. Much research work such as [7, 17] focuses on the privacy of personal data to ensure that the sensitive personal data processing takes place within the user's PDC instead of a third-party server.

### 2.3. The Classification of Personal Data

Researches have made elaborate classifications on personal data in terms of different criteria. This section summarizes previous work and gives a comprehensive classification. It is shown in Table 1.

The first dimension is the format which the personal data externally exhibits in, including documents, multimedia, web pages/fragments, email, and database. The second one is the source where the personal data is generated, including personal devices, web-based services, social networks, sensors, and the person himself. The third one is the abstraction level of personal data, including meta- and instance data. The fourth one is the semantics and functions and is the most complicated one, including the preference data, web footprints, and consumption and public service record. The last one is from the location where personal data is stored, including local/desktop data, distributed cloud based data, and centralized cloud-based data. Examples for each dimension are shown in the third column, with related literatures in the last column.

### 2.4. Views of Personal Data

Because of the complexity and the high volume of personal data, it is difficult to visualize the data all at once. Here we give five views to help decompose the whole personal data into small parts so as to achieve clearer visualization effects and better understanding on the data. It is called “*data projection*” being adopted in data visualization domain and so does in the personal data research. The five views are listed as follows.

1. *Time* (when): it organizes those personal data having a* timestamp* attribute in the form of the timeline. The unit of time might be a day, a week, a month, or a year, depending on the time granularity that the user is concerned about. Each data item is annotated onto the timeline in terms of the timestamp it owns, and the timelines will show different time granularity, for example, year, month, week, day, and so on. Data without any timestamp is not visualized. For example, a timeline is used to show the personal energy consumption of both household and business activities by the time view [37]. Further, personal data is classified into three tenses: past, present, and future.
2. *Location* (where): it organizes the personal data having a* location* attribute in the form of a geographical map. Many personal data have location-related attributes, so it is convenient to visualize the data on a map with latitude and longitude coordinates. For example, a world map is used to present the travelling location and route of users [37].
3. *People* (with whom): it organizes the personal data having some* socialization* attributes that direct to other persons. In other words, these data represent the user's social networking with others. Usually a directed graph is adopted to show the data projected in this view [4, 38].
4. *Belonging* (what): it views the personal data standing for a* virtual or physical belonging* of the user, for example, air miles, books, cars, and clothes. It is usually visualized in the form of a list.
5. *Finance* (how much): it views the data having some attributes with* economics significance*, that is, the data pointing to a specific financial transaction [10]. For example, a transaction record from* PayPal*, a purchase order from* Amazon*, and a credit card bill from* Citibank*. This view is usually visualized in the form of income and expenses curves.

**Figure 3:**
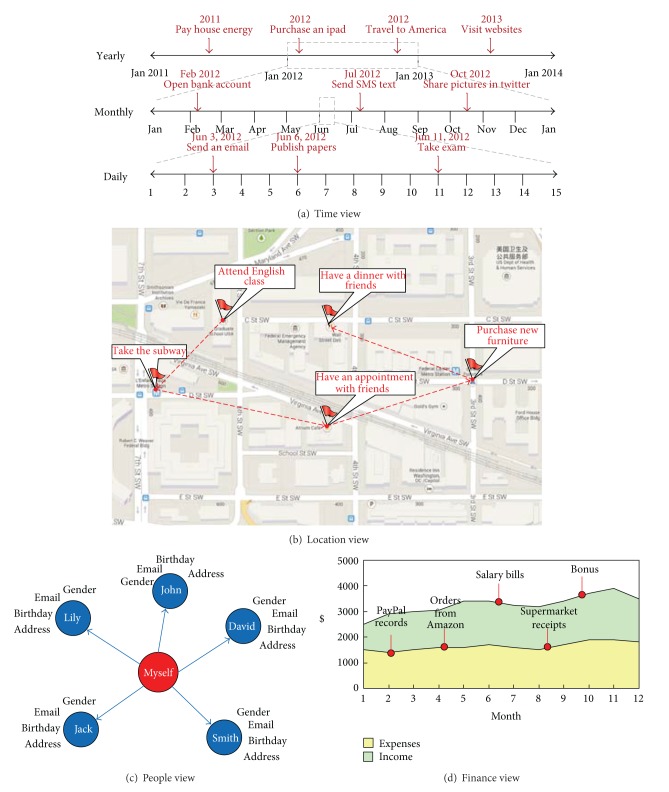
Personal data visualization for four different views.

It is important to note that each personal data item might fall into multiple views. For the purpose of personal data visualization, it is necessary to design for each view, and the combination of two different views, and so forth.  Figure 3 shows some examples of the personal data visualization, where Figure 3(a) is the time view, Figure 3(b) is the location view, Figure 3(c) is the people view, and Figure 3(d) is the finance view.

### 2.5. The Semantics Correlation between Personal Data

Although the personal data are aggregated from multiple services, they are inherently correlated by the user. This is called* data correlations*. For example, an activity “A business trip to Alaska for attending 2014 CLOUD conference” in* Google Calendar* is directly related to a flight order in* Expedia* and then related to a transaction record in* PayPal*, and so forth.

Data correlation will bring many benefits to the users. If we correlate personal data from various sources and link a wide variety of personal data in the web, the query efficiency could be speeded up [39]. Data correlations could be expressed in the form of static explicit declarations or in a relational data base system [40, 41].

But due to the high degree of dispersion of personal data, most of such correlations have disappeared. The recovery of semantics correlations after person data is collected is a challenging issue. Actually this is also the ideal of Semantic Web community and some feasible techniques such as Linked data have been put into practice in recent years.

### 2.6. Semantic Representation of Personal Data

Ontology and Linked data are the popular approaches for the semantic representation of personal data. Ontology defines a set of domain-specific concepts, attributes, and relations using a shared vocabulary [42]. An example is from [43] where a novel method is proposed to describe the metadata and instances of personal data in the form of ontology and provided an intelligent way to manipulate the data.

Linked data is an effective technique to interlink, share, and publicize various web resources by predefined ontology, built upon standard Web technologies such as HTTP, RDF, and URIs; thereby they can be automatically manipulated by computers. This enables personal data from different sources to be connected and queried efficiently, too [44].

## 3. Personal Data Cloud (PDC)

Based on the survey on personal data, we summarize the research progress on PDC and present a reference architecture of PDC.

### 3.1. Synonyms of PDC

PDC is a term proposed in this paper with the implications of collecting, aggregating, storing, indexing, correlating, and using the personal data. In the domain of personal data management, researchers focus on the same objectives but have employed different terms, such as the following.

- Personal Information Management (PIM) focuses on the acquisition or creation, store, organization, maintenance, retrieval, usage, and distribution of the personal information [45, 46].
- Personal Data Spaces (PDS) is an abstract data management technique aiming at personal data integration, based on existing matching and mapping generation techniques [17, 47].
- Personal Data Store (PDS), or called personal data vault or locker, is a service allowing an individual store, manage, and deploy their key personal data in a highly secure and structured way [5, 7, 48].
- Consumer-Centric Cloud Portal (C3P) is a middleware acting as an intermediary between Apps and services and assists Apps access the personal data in cloud in a device-, time-, and location-independent way [49].
- Personal Cloud Butler (PCB) is a service that provides a safe haven for personal digital assets and supports sharing with fine-grain access control [50].
- Personal Cloud (PC) is a similar service allowing users access their personal data across multiple devices [51].

The reason why we use Personal Data Cloud (PDC) to unify this miscellaneous terms listed above is straightforward: firstly, the managed object is “*Personal Data*;” secondly, the management of personal data is more inclined to a centralized* cloud* environment; thirdly, the management issues should cover the full lifecycle of personal data.

### 3.2. Reference Architecture of PDC

Essentially, a PDC is a SaaS application deployed on a cloud. A reference architecture is necessary for PDC developers to plan its main components and their interconnections.

**Figure 4:**
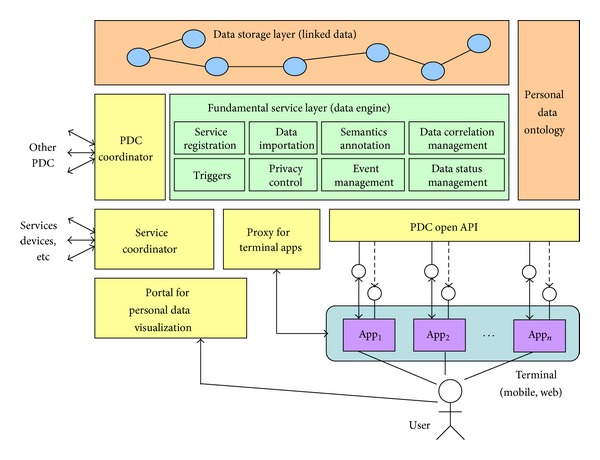
A reference architecture of PDC.

As shown in Figure 4, PDC has a multilayer architecture supporting the seamless integration between the Apps installed on mobile terminals and a set of PDC services deployed on the cloud. This architecture is proposed by the synthesis of the personal data management frameworks presented by the literatures mentioned in Section 3.1.

Here we give a brief introduction to each layer.

1. *Personal Data Ontology*. It is an extensible ontology defining a set of standard terms (classes, attributes, and relations) that covers various service domains. It offers the abstraction of various types/sources of personal data and is intended to be completely independent of the physical representation of personal data.
2. *Data Storage Layer*. It is a centralized data repository where the meta and instances of the personal data are centrally stored. Either the metadata or the instance data is annotated by the Personal Data Ontology so that their semantics is unified and the potential semantics relations are recovered. All the data is indexed and represented in the form of Linked Data which facilitates the convenient query and navigation.
3. *Fundamental Service Layer (Data Engine)*. It is the core of PDC and composed of a set of fundamental services.
  - *Service Registration* component allows users to register the services they are using to PDC so that the personal data that these services produce is to be imported to PDC for the unified management.
  - *Data Importation* component enables the (semi-) automatic importation of the personal data produced by the registered services into PDC and the data synchronization between services and PDC if the same data in either side was updated.
  - *Semantic Annotation* component is to establish the semantics mapping between the metadata imported from services with the Personal Data Ontology for semantics unification.
  - *Data Correlation* component is to manage the semantics correlations between personal data produced by different services so that they are represented as Linked Data with the help of Personal Data Ontology.
  - *Privacy Control* component is used to set up the privacy rules/policies on the personal data, for example, what classes, attributes, and relations could be accessed by which of the external services and which of the other users, thereby protecting the data privacy. This issue is to be discussed in Section 3.3.
  - *Data Status Management*,* Event Management,* and* Triggers* are the three components enabling the PDC-based service collaborations. Data Status Management is responsible for monitoring the dynamic changes of personal data and then generating the corresponding events; Event Management consolidates all the generated events in a queue; and Triggers try to identify the potential collaborations, distribute the related events to external services or mobile apps, and then trigger the collaborations.
4. *PDC Coordinator*. As each user has his own PDC, the PDC coordinator enables the communication between multiple PDCs so that the social service collaborations between different users are established.
5. *Service Coordinator*. It is responsible for the coordination between the services that have been registered to PDC when the Trigger component identifies the potential collaborations between them.
6. *Proxy for Terminal Apps*. The potential collaboration occurs not only between services, but also possibly between the apps in mobile terminals. Each terminal app has a proxy on PDC and could be triggered by the proxy through* callback* mechanism. In other words, a change of personal data would lead to the execution of some actions offered by the apps.
7. *Open API*. It facilitates bidirectional data exchange between PDC and various terminal apps, allowing the apps access the data in PDC in a standard way.
8. *Portal for Personal Data Visualization*. It is a GUI where users browse and query their personal data in selected view(s) and tense(s) (discussed in Section 2.4). Data is graphically visualized.
9. *Apps*. This refers to the various terminal apps.

It is noted that not all above components have been implemented by existing works. The PDC architecture is still an open issue both in research and practice.

### 3.3. An Operational Scenario of the PDC Reference Architecture

To illuminate how the PDC reference architecture works, here we give an operational scenario. Suppose there are two users named Jack and Lily who have their own PDC, and they use a set of services including* TypoWeather* (a weather forecast service),* EatThisMuch* (an automatic diet planner service),* MyClean* (a maid cleaning service),* HealthLoop* (a medical service to monitor and communicate with patients during the recovery process),* ReviewsTalk* (a customer review service),* Gmail*,* Twitter*,* Facebook*,* Amazon*,* Dropbox*, and* Paypal*, and four mobile apps including* CityMapper* (a transport app),* DoctoronDemand* (a* talk-to-a-real-doctor* app),* MapMyNearest* (a local service search app), and* HailoCab* (a taxi app).

Firstly, Jack and Lily register the services and apps that they are using into their own PDC by the service registration component. Then, the PDC imports their personal data from these services/apps by the data importation component. After the importation, each personal data item is annotated to the Personal Data Ontology by the semantics annotation component, and the potential semantics correlations between different personal data items are recovered by the data correlation component. For example, the review data in* ReviewsTalk* is annotated by the ViewPoint class in the PDC ontology, the purchasing order data in the* Amazon* is annotated by the Order class, and the two data items are correlated together indicating that Jack bought clothes from* Amazon* and commented it on* ReviewsTalk*. All the personal data is stored in the Data Storage Layer in the form of linked data. Jack can set up his privacy rules by the privacy control component, for example, whether his personal data generated by* ReviewsTalk* could be accessed by other services such as* Twitter* and* DoctoronDemand*, and by other users such as Lily.

If Jack bought a new laptop from* Amazon*, then a new order data will be automatically imported into his PDC. The data status management component automatically identifies the new data; then the event management component generates a new event, and the trigger component plans the potential invocations of other services (e.g., to post a microblog on* Twitter*). It is the service coordinator component that is responsible for the real invocations.

If Lily would like to invite Jack to come to her birthday party, she accesses Jack's PDC to get his food preference; then buys a birthday cake by the* MapMyNearest* app on own mobile phone. Lily and Jack will use* HailoCab* app to taxi to the place of the party. In this collaborative process, the PDC coordinator component is responsible for the collaborations between Jack and Lily's PDC, and the service coordinator component and the proxy for terminal apps are responsible for the invocations of the corresponding services and apps of Jack and Lily, respectively.

### 3.4. The Privacy and Security Management of PDC

Data privacy and security are always boring issues, especially because the personal data that has higher degree of privacy because they represent the history of a person. Accidental disclosure and misusing of personal data would result in serious consequences.

PDC researchers are working on this issue from two levels: policy level and infrastructure level. The former aims at defining privacy policies and the latter aims at reasoning and executing the predefined privacy policies. For example, policy ontology is used to determine whether the requester has the permission to access the data based on data owner's (or provider's) privacy policies, and a reasoning engine performs the reasoning over the privacy policies for actual control [52]. Privacy-Lookout (PL) [16] is another work to allow people to be on the lookout for transgressions of their personal data privacy semantically enriched on the metainformation of the personal data.

There are three types of privacy management that should be emphasized in PDC. The first type is user-oriented privacy, that is, the data owner determines which personal data items are open to which users, and these users are authorized with the permission of acquiring these data. For example, a friend could discover if a house owner is away, while a complete stranger might only see the phone number, and a colleague might not see the personal phone number but only the professional number and professional blog [7]. The second type is service-oriented privacy, that is, the data owner determines which personal data items are open to which services that he is using. This indicates that, although these services should be independent of the PDC of a user, they could be authorized to acquire the user's personal data to enhance their own functions and consequently improve user experiences, although these data are produced by other services [17]. The third type is the PDC provider oriented privacy; that is, there is latent threat when it comes to the data loss or leakage which may be committed by malicious PDC Providers [53]. This is a common problem for all cloud services. A basic solution is to encrypt personal data using a user-centric key management scheme [53], and more advanced security control mechanisms include a trust enhanced secure cloud storage service named TS3 [54] and SafeShare which encapsulates personal data in self-controlling objects (SCO) and monitors the operations of any other users [55].

## 4. Existing Commercialized Products and Prototypes of PDC

Well-known IT companies such as* Microsoft*,* Google*, and* EMC* have offered many online services in PDC domain, for example, MediaFire, SkyDrive, Evernote, Google Drive, and DropBox, and so forth. Nevertheless, most of these commercialized services focus only on one type of personal data, especially the* file-based* personal data. In other words, they look more like the cloud-based personal disk with almost infinite storage and limited data-sharing with others. This is not the ultimate goal of PDC.

In research, some researchers have developed several PDC prototypes but have not yet been put into practical use. Here we introduce some examples.

1. *di.me* [43] is a distributed personal information sharing system. The extracted information and observed personal activities are exploited to automatically recognize personal situations, provide privacy-related warnings, and recommend and/or automate user actions.
2. *Personal-Cloud Butler *(*PCB*) [50] is a decentralized infrastructure that lets users participate in online social networking without loss of data ownership. It has a person-centric architecture, and each individual uses a Personal-Cloud Butler (PCB) service that provides a safe haven for one's personal digital assets and supports sharing with fine-grain access control.
3. *Personal Cloud Platform *(*PCP*) [6] is a platform for the management of service clouds providing the user with a unified environment for handling his activities and collaborations. Within a personal cloud, the PCP enables the definition of global collaboration groups and a holistic management of the workspace awareness, concerning all the integrated services.
4. *Personal Information Management *(*PIM*) [56] is a tool supporting the lightweight, user-driven mixing of previously unintegrated data, with the objective of allowing users to take advantage of the emerging ecosystems of structured data currently becoming available.
5. *Open Personal Data Store *(*openPDS*) [17] is a system for managing the personal information that is organized by Linked data and allowing users to collect, store, and give fine-grained access to their data in the cloud. It also protects users' privacy by privacy-preserving group computations to aggregate data across users without the need to share sensitive data with an intermediate entity.
6. Privacy-Lookout (PL) is a semantic web-based framework allowing people to be on the lookout for transgressions of their personal data privacy with respect to their privacy principles [16]. To achieve this objective, a personal linked-data view is created and the meta-information of the personal data existing in the Web is semantically enriched.

Other prototypes include Memoria-Mea [57] and Menagerie [58].

Table 2 gives a brief comparison between these existing PDC prototypes/systems from four perspectives. It is observed that most of them cannot cover all the aspects of PDC, and there is still a lot of work to do.

## 5. Open Issues on PDC Research

The research of PDC is in rapid progress but is far more mature. Here we list five open issues to be carefully addressed in the future.

**Figure 5:**
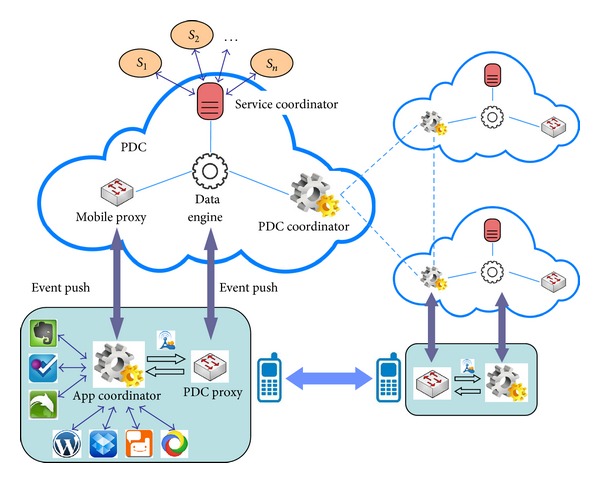
PDC-based adaptive service collaboration.

**Figure 6:**
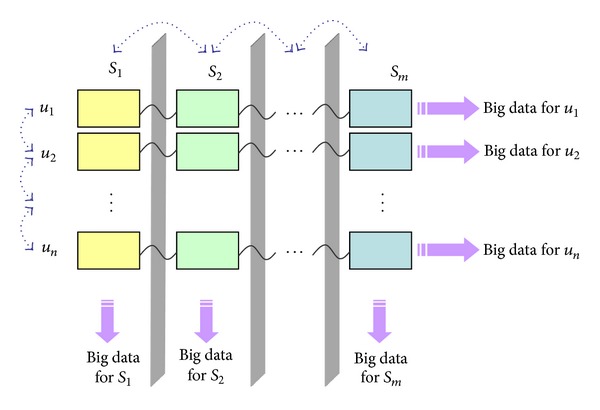
Service-oriented versus user-oriented big data.

1. Automatic collection of personal data. Data collection methods adopted in current research look obsolete. Typical methods include (a) API-based method, (b) web crawler based method, and (c) manual importation by users. The first one is limited because some services do not provide open APIs, or the data acquired from the APIs are incomplete. The second one is quite time-consuming because the format of those web pages where personal data is contained is diverse, and many privacy personal data are not in public web pages or some services do not allow their web pages to be crawled. The third one is time-consuming too, and it is difficult to keep the synchronization between the original data source and PDC.
2. Semantics unification of personal data. Although ontology and Linked Data have been widely adopted for this issue, the existing ontology still focuses on limited domains; however, services that people use are very diverse and span multiple domains. A universal ontology is urgently required.
3. PDC-oriented programming model for mobile apps and cloud services. Being a continuity of the first issue, this one is to invent a new programming model so that the services/apps around a person have the capacity of automatically synchronizing the personal data with users' PDC. There are four key enhancements on current programming models of cloud services and mobile apps.
  - The service should allow a user specify the address of his own PDC so that it could synchronize the personal data it generates or updates to the PDC in real time.
  - The service should support the universal ontology and map the personal data it generates to the standard ontology so that other services/apps can understand the semantics of its data.
  - The service could acquire from the user's PDC the personal data that is originally generated by other services to enrich its functionality.
  - The service should offer callback interfaces which are to be invoked by the PDC's service coordinator component so that it is automatically coordinated with other services/apps.
4. PDC-based adaptive service collaboration. This is the biggest unsolved issue, indicating that services around a person should dynamically and adaptively collaborate with each other so that users' personalized requirements are fulfilled. As mentioned in Section 1, now different services are isolated and the collaborations between them are usually conducted by service brokers instead of the user. Under the support of PDC, user-centric adaptive service collaboration becomes possible.  Figure 5 shows the basic collaboration mechanism. The data engine in PDC is responsible for monitoring the dynamic changes of personal data and adaptively planning the potential collaboration relationships among services, and the service coordinator harmonizes the execution of the collaboration via the callback interfaces of related services. If the collaboration requires the participation of mobile apps, the mobile proxy pushes the messages to the mobile where the app coordinator is responsible for coordinating the invocation and execution of related apps which synchronize the personal data with the PDC's data engine during the execution. Another adaptive collaboration happens among different users, and it is the PDC coordinator that is responsible for harmonizing such collaboration.
5. User-centric big data. Big data is an extremely hot topic but usually focuses on service-oriented big data. As shown in Figure 6, due to the isolations between services, big data owned by an organization cannot be aggregated with other big data. In PDC scenario, the big data is user-oriented; that is, all the data in PDC are related to the same person, no matter which service produces the data. Analyzing and mining user-oriented big data would bring about more significance because these data integrate dispersed fragments of personal history and contain richer knowledge.

## 6. Conclusions

This paper makes a short survey on personal data management and Personal Data Cloud (PDC) based on the summarization of literatures published in recent years. As an emerging and significant issue, user-oriented big data has showed great power on various applications; therefore, the uniform and centralized personal data management is urgently required. Current popular PDC products are far from encouraging (i.e., focusing only on file-based cloud storage), and on the other hand, most of the existing PDC prototypes have not been yet put into practice due to some open issues. We do hope the clarification of PDC's state of the art will motivate researchers work in more depth on the open issues listed in this paper.

## Figures and Tables

**Table 1:** Multidimensional classification of personal data.

Dimension	Categories	Examples	References
Format	(1) Document	DOC, PPT, spreadsheets, and so forth	[3–5, 9, 10, 12, 13, 18]
	(2) Multimedia	Images, videos, audio, and so forth	
	(3) Web page and fragment	Search keywords, visited links, cookies, and so forth	
	(4) Email	Gmail, Yahoo! Mail, and so forth	
	(5) Database	Business data stored in domain-specific services, such as orders, calendars, wish lists, and so forth	

Source	(1) Personal devices	PC, smart phones, mobile devices, tablet, and so forth	[5–8, 14, 19–22]
	(2) Services	Web applications, and so forth	
	(3) Social network	Facebook, Twitter, blogs, and so forth	
	(4) Sensors	GPS, thermometer, wearable devices, and so forth	
	(5) The person himself	Email, work schedule, documents, pictures, video, audio	

Abstraction level	(1) Metadata	The descriptions of personal data	[11, 14–17]
	(2) Instance data	The contents (instances) of the metadata	

Semantics and functions	(1) Preference data	Preferences on books, music, cities, friends, wish list, and so forth	[19, 23–32]
	(2) Communication record	SMS text, phone records, address book, and so forth	
	(3) Web footprints	Visited websites, search keywords, social comment logs and social graph, and so forth	
	(4) Personal profile	Height, weight, published papers, education/career experiences, exam performance, and so forth	
	(5) Consumption service record	Bank account and transaction records, flight and hotel orders, car rental orders, supermarket records, e-commerce transaction record, and so forth	
	(6) Public service record	Personal salary records, household energy record, personal credit, and so forth	

Storage location	(1) Local/desktop storage	Files located on personal computers and devices	[20, 33–36]
	(2) Distributed cloud storage	Data stored in the cloud of a service	
	(3) Centralized cloud storage	Many personal data centralized stored in a public cloud	

**Table 2:** Comparison between existing PDC prototypes/systems.

PDC prototypes	Data collection from services	Personal data storage	Semantics unification	Privacy and access control	Open API for personal data access	Personal data visualization	Supporting service collaboration
*di.me *	Automatic	File	Ontology	YES	NO	YES	NO
*PCB *	Semiautomatic	File	RDF & ontology	NO	YES	NO	NO
*PCP *	Automatic	N/A	N/A	YES	NO	NO	YES
*PIM *	Semiautomatic	File	Ontology	NO	NO	NO	NO
*openPDS *	Semiautomatic	File	N/A	YES	NO	YES	NO
*PL *	Automatic	File	Linked data	YES	NO	NO	NO
*PC *	Automatic	File	N/A	NO	YES	YES	NO
